# Selective Screening Strategies for Gestational Diabetes: A Prospective Cohort Observational Study

**DOI:** 10.1155/2017/2849346

**Published:** 2017-10-22

**Authors:** Sumaiya Adam, Paul Rheeder

**Affiliations:** ^1^Department of Obstetrics and Gynecology, University of Pretoria, Pretoria, South Africa; ^2^Department of Internal Medicine, University of Pretoria, Pretoria, South Africa

## Abstract

**Aim:**

We aimed to develop a prediction model for the diagnosis of gestational diabetes and to evaluate the performance of published prediction tools on our population.

**Methods:**

We conducted a cohort study on nondiabetic women < 26 weeks gestation at a level 1 clinic in Johannesburg, South Africa. At recruitment, participants completed a questionnaire and random basal glucose and HbA1c were evaluated. A 75 g 2-hour OGTT was scheduled between 24–28 weeks gestation, as per FIGO guidelines. A score was derived using multivariate logistic regression. Published scoring systems were tested by deriving ROC curves.

**Results:**

In 554 women, RBG, BMI, and previous baby ≥ 4000 g were significant risk factors included for GDM, which were used to derive a nomogram-based score. The logistic regression model for prediction of GDM had *R*^2^ 0.143, Somer's Dxy rank correlation 0.407, and Harrell's c-score 0.703. HbA1c did not improve predictive value of the nomogram at any threshold (e.g,. at probability > 10%, 25.6% of cases were detected without the HbA1c, and 25.8% of cases would have been detected with the HbA1c). The 9 published scoring systems performed poorly.

**Conclusion:**

We propose a nomogram-based score that can be used at first antenatal visit to identify women at high risk of GDM.

## 1. Introduction

Gestational diabetes mellitus (GDM) is regarded as glucose intolerance with first onset in pregnancy. The diagnosis of GDM infers an increased risk of both short- and long-term adverse outcomes for the mother and fetus [1]. The current guidelines of the International Federation of Gynecology and Obstetrics (FIGO) recommend universal screening of pregnant women for GDM with a 75 g 2-hour oral glucose tolerance test (OGTT) [2]. The lower thresholds recommended by FIGO are derived from the findings of the Hyperglycemia and Adverse Pregnancy Outcomes (HAPO) study. The HAPO study found that the adverse events associated with GDM increase along a continuum with increasing hyperglycemia [3]. The estimated prevalence of GDM based on the FIGO guidelines varies between 11.1–44.3% [4, 5].

Universal screening for GDM has the advantage that all pregnant women are screened as part of routine antenatal care. However, this will place an added burden, both financial and personnel, on the health care system. Selective screening based on risk factors such as advanced maternal age, obesity, family history of diabetes, and previous adverse pregnancy outcomes such as recurrent or unexplained pregnancy losses, large-for-gestational-age babies, or congenital abnormalities has been proposed as a screening strategy for GDM. Selective screening based on risk factors performs poorly as a screening tool with up to one-sixth of women with GDM diabetes being missed [6]. Furthermore, recall of historical risk factors is poor, medical records are often incomplete or unavailable, or the recorded history is not often considered by the clinical staff to trigger screening for GDM. Thus, the current risk factor-based screening is ineffective.

Whilst the traditional risk factor-based screening program performs poorly for the screening of GDM, there are several published risk-scoring systems that hold promise. These models combine maternal characteristics and medical history into a simple clinical prediction tool. This may assist in identifying women who require an OGTT with greater efficacy, accuracy, and efficiency [7–15]. However, these models were developed on non-African populations in tertiary centres using data obtained from a selective screening approach in most instances [7–15].

The purpose of this study was to develop a clinical prediction model for GDM in a South African population and to evaluate the performance of the published prediction tools on our study population. The introduction of such a prediction tool would reduce the number of OGTTs, hence decreasing the workload and financial burden on an overburdened healthcare system.

## 2. Materials and Methods

This paper forms part of a larger study investigating screening strategies for GDM in a South African population. We carried out a prospective cohort observational study at a level 1 primary healthcare clinic in Johannesburg. One thousand consecutive pregnant women that were less than 26 weeks pregnant were recruited. Patients known with diabetes mellitus or greater than 26 weeks pregnant were excluded [16].

At recruitment, each patient completed a questionnaire including demographic data and an evaluation of risk factors for GDM. Risk factors considered were obesity, that is, a body mass index (BMI) ≥ 30 kg/m^2^, age ≥ 35 years, a family history of diabetes mellitus, a history of a delivery of a baby ≥ 4000 g in a prior pregnancy, glycosuria, a history of GDM in a prior pregnancy or a history of a baby with a congenital abnormality, and an unexplained stillbirth or recurrent pregnancy losses. Gestational age was based on the patient's last normal menstrual period, ultrasound-determined gestation, or by measuring of the symphysis to fundal height.

A random blood glucose (RBG) and glycated hemoglobin (HbA1c) levels were measured at recruitment on a Roche Accu-check Active point-of-care device and at the laboratory. The glucometer was regularly calibrated as per manufacturer guidelines, and glucose was measured on whole capillary blood. Measurements on the glucometer were not affected by haematocrit. If the random glucose was greater than 11.1 mmol/l or HbA1c was greater than 6.5%, the patient was referred to the local hospital for further management of overt diabetes mellitus. Else, a 75 g two hour oral glucose tolerance test (OGTT) was scheduled for between 24 and 28 weeks gestation. GDM was diagnosed based on the FIGO criteria [2]. All blood was drawn by a registered nurse and was stored on ice until it was delivered to the laboratory on the same day. Point-of-care tests were performed on-site.

We used R version 3.3.0 [17] with packages PredictABEL [18] and rms [19]. In order to compare the different prediction models, we imputed missing values using multivariate imputation by chained equations [20]. Because our dataset is large enough, we used all clinically relevant predictors in a logistic regression model [17]. Random serum glucose demonstrated a nonlinear relationship with the log odds of the outcome, and we used a restricted cubic spline with two knots in the model. The other continuous variables were not transformed in the model. In order to determine the degree of optimism with this model, we validated and calibrated the model with 200 bootstrap samples according to Harrell [19].

To reduce the number of variables for a more parsimonious model, we used the method of approximation as suggested by Harrell (to remove the variables that would have the smallest effect on the *R*^2^ coefficient of determination of the linear regression model of variables on the linear predictor of the logistic regression model as the outcome) [19]. We tested the interaction of HIV with the other variables.

We then investigated the effect of having a model with and without HbA1c as some centres may and some may not have HbA1c testing readily available. These models were compared using Harrell's c-index, Somer's Dxy rank correlation, the Brier score, *R*^2^, and the net reclassification index (NRI) (at probabilities of 10, 50 and 100%) as well as the integration discrimination improvement (IDI) [17]. Harrell's c-index (c-index > 0.5 shows good predictive ability) and Somer's Dxy (where Dxy = 1 when the model is perfectly discriminating) are measures of the general predictive power of a regression model. In effect, they are a natural extension of ROC curve areas. The Brier score (where the best possible score is 0 for total accuracy) measures the accuracy of probabilistic predictions, that is, it is the average gap between the calculated probability and the actual outcome. *R*^2^ (where 1 fits the regression line perfectly) provides information on the goodness of fit of the model. The NRI is an index of how well a new model classifies subjects (in this case how well does our prediction model identify patients at high risk of GDM). The IDI similarly is a tool that evaluates the capacity of a marker or model to predict the outcome (i.e., how well can the prediction model identify patients with GDM).

Categorical NRI equal to *x*% means that compared with individuals without outcome, individuals with outcome were almost *x*% more likely to move up a category than down. The function also computes continuous NRI, which does not require any discrete risk categories and relies on the proportions of individuals with outcome correctly assigned a higher probability and individuals without outcome correctly assigned a lower probability by an updated model compared with the initial model. IDI equal to *x*% signifies that the difference in average predicted risks between the individuals with and without the outcome increased by *x*% in the updated model. Finally, for ease of use, nomograms were generated for the model with and without HbA1c [19].

Furthermore, nine published risk prediction models for GDM were identified [7–15]. The risk prediction model from each study was applied to our study population, and a receiver operating curve (ROC) was generated to evaluate its performance as a screening tool. We thereafter compared the two ROC curves derived from independent samples to assess if there was a significant difference in the area under the curve.

Approval for this study was obtained from the University of Pretoria, Faculty of Health Sciences Ethics Committee (Protocol 180/2012) and was performed in accordance with the 1964 Declaration of Helsinki and its amendments. Informed consent was obtained from every patient prior to entry into the study.

## 3. Results

One thousand (1000) pregnant women were recruited. Eighty-two (8.2%) women had fetal losses and did not continue with the study, 163 (16.3%) women moved away from the area and were thus lost to follow-up, 194 (19.4%) women were unreachable, and seven (0.7%) women withdrew consent for the study. Thus, 554 (55.4%) women had complete data available for analysis. One hundred and forty-four (25.8%) women had GDM.

The mean age of the population was 27.2 years (IR 13–42 ± 5.8), parity 1.1 (IR 0–5 ± 1), BMI 26.7 kg/m^2^ (IR 22.7–47.2 ± 5.4), random glucose 4.5 mmol/l (2.9–9.3 ± 0.7), and HbA1c 5.2% (33.3 mmol/mol) (IR 3.8–6.5% ± 0.4; 18–47.5 mmol/mol). One hundred and sixty (28.9%) women were HIV positive of which 59 (36.9%) were on highly active antiretroviral therapy (HAART), 78 (14.1%) women had a positive family history of diabetes mellitus, 55 (9.9%) had a history of a previous stillborn or congenitally abnormal baby, and 44 (7.8%) women had previously delivered a baby > 4000 g.

We considered the role of the random glucose (AUROC 0.63, 95% CI 0.58–0.68) as a screening tool. Furthermore, we included clinically relevant predictors, namely, delivery of a previous baby ≥ 4000 g, random glucose, BMI, family history of diabetes mellitus, HbA1c, history of a previous stillbirth, or previous baby with a congenital abnormality and age, in a logistic regression model [2]. The odds ratio of the full model is illustrated in Supplementary Figure 1 (S1 odds ratio of full model (continuous variables: 75th versus 25th percentile) available online at https://doi.org/10.1155/2017/2849346). The performance of this model including all clinical variables is illustrated in Table 1.

The model was then validated and calibrated with 200 bootstrap samples according to Harrell [19]. One can observe that at higher predicted probabilities the actual probabilities are less in the optimism-corrected model indicating a fair degree of optimism as demonstrated in Supplementary Figure 2 (S2 correcting the model for optimism). At higher predicted probabilities, the actual probabilities are less in the optimism-corrected model indicating a fair degree of optimism. The discriminatory indices for the validated model are illustrated in Table 1. The quantile absolute error was 0.029. The slope of the bias-corrected model was 0.870, it has a 0.130 difference when corrected for optimism.

In order to get a smaller model, we used an approximation method as suggested by Harrell [19] to remove variables that would have the smallest effect on *R*^2^ of the linear regression model (Table 2). We removed family history of diabetes mellitus, history of a previous stillbirth or previous baby with a congenital abnormality, and age and still maintained 95.7% of *R*^2^.

We analysed the interaction of HIV and HAART with the clinical variables. HIV nor the HAART had any interaction with a history of a delivery of a previous baby greater than 4000 g (*p* = 0.066), the random glucose (*p* = 0.835), or BMI (*p* = 0.801). Overall, HIV nor HAART had an effect on our proposed model (*p* = 0.974)

We thereafter used a smaller model to determine whether adding HbA1c would add any predictive value to the model as demonstrated in Supplementary Figure 3 (S3 comparison of predictive value of model with and without HbA1c). We found that adding an HbA1c did not significantly improve the predictive value of the model (Table 2). The slope of the bias-corrected model was 0.933, and it has a 0.0.067 difference when corrected for optimism.

By adding the HbA1c to the model 12%, 4%, and 9% of patients will be downclassified to being at low risk of GDM at 10, 50, and 100%, respectively. Similarly, 0%, 4%, and 0% will be upclassified to being at high risk of GDM at 10, 50, and 100%, respectively. The integrated discrimination improvement (IDI) shows that the discrimination slope of the updated model with the added HbA1c was 10.8% higher than the original model.

The le Cessie-van Houwelingen-Copas-Hosmer unweighted sum of squares test for global goodness of fit for the model with HbA1c gave a *p* value of 0.87 and for the model without HbA1c 0.81.

**Figure 1:**
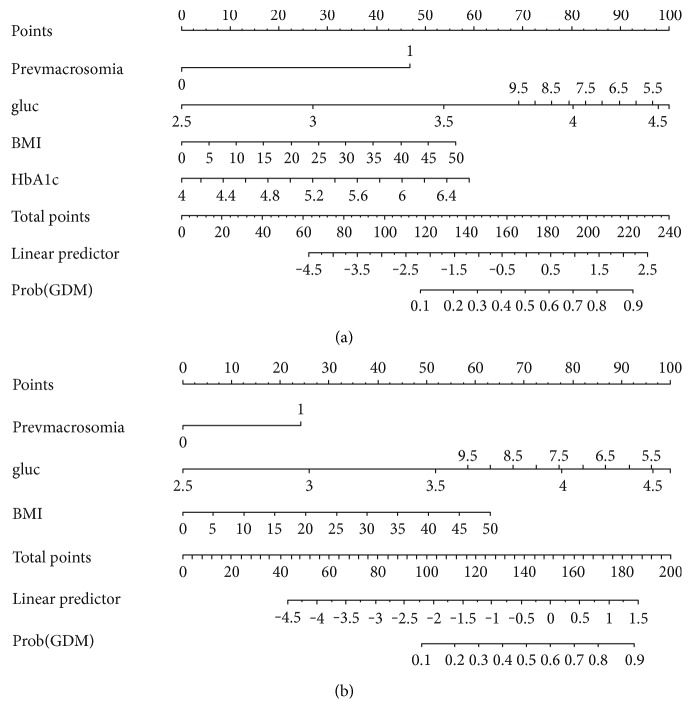
Nomograms (a) with HbA1c and (b) without HbA1c. The nomograms consider the history of delivery of a previous baby > 4000 g (prevmacrosomia: 0 = no, 1 = yes), random glucose (gluc: measurement in mmol/l), and BMI (BMI: mass in kilograms/height in metre^2^). Two nomograms are illustrated to show the difference with and without and HbA1c measurement being included. The score is derived by aligning the points on each number line with the “points” line at the top. The total score is then calculated and plotted on the “total points” line. When this total score is compared to the prob(GDM) line, the probability of developing GDM is derived. For example, a 30-year-old woman who is now para 2 gravida 3, with a BMI of 35 kg/m2, who previously delivered a 4.3 kg baby, has an HbA1c of 5.8% and now has a random glucose of 6.7 mmol/l will have a score of 155 and thus a 50% chance of developing GDM in this pregnancy based on the nomogram without the HbA1c. Her score is 182 and thus a 52% risk of developing GDM if the HbA1c is incorporated into the prediction model. Prevmacrosomia: history of delivering a baby > 4000 g; gluc: random glucose; BMI: body mass index; HbA1c: glycated hemoglobin; Prob(GDM): probability of developing gestational diabetes.

Finally, for ease of use, nomograms were generated for the model with and without the HbA1c (Figure 1).

We then compared the efficacy of the nomograms at >10% and >15% probabilities of GDM (Table 3) as we will need to establish the cutoff risk above which a woman is deemed at high risk of developing GDM. At a cutoff of 10%, 58 (10.5%) and 50 (9.0%) fewer OGTTs would be carried out if HbA1c was or was not incorporated into the nomogram, respectively. Two (0.4%) and one (0.2%) cases of GDM would be missed if the nomogram with and without the HbA1c was applied, respectively. Similarly, at a 15% cutoff, 125 (22.6%) and 103 (18.6%) fewer OGTTs would be carried out if HbA1c was or was not incorporated into the nomogram, respectively. Nine (1.6%) cases of GDM would be missed whether or not HbA1c was incorporated into the nomogram.

Table 4 demonstrates the performance of each published prediction model for GDM once it was applied to our study population as compared to the population that it was derived from.

## 4. Discussion

This study aimed to evaluate the use of risk indicators to develop a statistical prediction model for GDM. Traditionally identified risk factors such as BMI, age, or family history of diabetes mellitus have been associated with GDM in other populations [21–24]. Data on GDM in Africa, especially since the introduction of the FIGO criteria, is scant. Available data found an association with GDM and obesity, family history of diabetes mellitus, previous stillbirth, previous macrosomic child, and age > 30 years in some sub-Saharan African populations [23].

The fasting glucose appears to be a very attractive tool for screening pregnant women for GDM. However, this would necessitate that all pregnant women present in a fasted state for screening, thus this can only take place on the second antenatal visit and would require all pregnant women to be tested. While this may not seem unrealistic, it can prove to be challenging in a low-income country where women have to travel a great distance to the healthcare facility and they often do not have funds for transport. Thus, we investigated an alternate screening tool that could be used easily on the first antenatal visit to stratify a women's risk for GDM in the current pregnancy.

We found that a previous history of delivering a baby weighing ≥ 4000 g and an elevated random blood glucose were independent predictors of developing GDM. Church et al. and Meek et al. found that the random glucose was a promising screening tool for GDM with AUROC of 0.8 and 0.86, respectively [25, 26]. By comparison, these retrospective studies employed a 2-stage screening protocol for GDM and did not use the currently widely accepted FIGO diagnostic criteria. By comparison, the basal random glucose alone was a poor predictor of women likely to develop GDM in our study. Thus, we propose that our scoring system, by adding other variables to the random blood glucose, will better identify women at risk at GDM compared to the random blood glucose alone. This premise requires prospective validation.

Other studies have also identified risk factors [19–24]. Only nine of these studies summarised this into a score or a clinical prediction model, of which we were able to test eight on our study population (Table 4). These tests performed poorly as a screening tool in our study population compared with their derivation populations. This may be a result of us testing these scores on a low-risk pregnant population. Risk factors may play a less significant role in predicting GDM when universal screening is applied, and the FIGO diagnostic criteria are used. By contrast, most of the aforementioned scoring systems used a selective screening approach and used criteria other than that recommended by FIGO for the diagnosis of GDM in the derivation and the validation of their scores [7–14]. Furthermore, many of these scores use logarithmic equations in their calculations, thus necessitating a computer in the clinic, which is not always available in South African antenatal clinics [9, 13–15].

As South Africa is a resource-restricted country that faces a dual burden of disease, that is, communicable and noncommunicable diseases, a selective screening approach is an attractive option for the diagnosis of GDM as it seems the more cost-effective approach. As a risk-factor-based approach performs inconsistently, a scoring system that incorporates the more significant risk factors in a population may be a better option. We have proposed a nomogram that incorporates the significant factors in a South African population. The BMI and history of previous deliveries are currently part of routine antenatal practice. The random blood glucose can easily be tested at the first antenatal visit, making this a clinically applicable tool for early pregnancy. In some settings, an HbA1c may be available. However, we have demonstrated that including the HbA1c into the risk stratification tool does not significantly affect the patient's risk of GDM. South Africa has a high burden of HIV. In our study, HIV did not affect the incidence nor did it contribute as a predictive marker of GDM.

The risk stratification system proposed by Harrison et al. performed well in our study population [12]. This score is based on the patient's age in years, BMI, race, family history of diabetes mellitus, a history of GDM in a prior pregnancy, and fasting plasma glucose. The advantage of this system is that it is a scoring system rather than a logarithmic equation, and it incorporates information that is routinely obtained on the first antenatal visit. However, it will require the patient to present to the clinic in a fasted state for a second visit before the risk stratification can be completed. This may be problematic in our setting of a low-middle income population that may not live close to the clinic. In addition, this selective screening approach may not be complied with if it requires the healthcare worker to recalculate the risk on multiple visits. Our proposed nomogram incorporates only two historical factors and a random glucose, thus making it quick and easy to use at the first antenatal visit.

There is an increasing health burden related to obesity and type 2 diabetes mellitus in sub-Saharan Africa, yet little is known about the prevalence of GDM [27]. In South Africa and many other countries worldwide, screening programs are based on risk factors. However, it has been demonstrated that this approach shows a low compliance to guidelines. Hence, many women are not screened and GDM remains underdiagnosed [28]. The FIGO criteria have been criticised for its low diagnostic thresholds. Several studies have shown that women diagnosed with GDM based on the IADPSG criteria had higher adverse outcomes such as fetal macrosomia, risk of primary caesarean delivery, and preeclampsia compared with women with no GDM [29, 30].

Screening for GDM is necessary as it has both short- and long-term implications for the mother and child. The FIGO diagnostic criteria have been adopted almost universally [2]. We have proposed a simple nomogram that can be used for predicting the probability of developing GDM at the first antenatal visit. A limitation of this study is that the prediction model needs to be tested prospectively in the screening and diagnosis of GDM so that it can be validated, and a threshold of clinical usefulness can be determined before it can be widely implemented.

## Supplementary Material

Figure S1. Odds ratio of full model (continuous variables: 75th versus 25th percentile). Figure S2 Correcting the model for optimism. Figure S3. Comparison of predictive value of model with and without HbA1c.

## Figures and Tables

**Table 1:** Discrimination indices of the full predictive model including all clinical risk factors.

Discrimination index	Full model with all variables	Validated model
*R* ^2^	0.143	0.109
Harrell's c-index	0.703	
Somer's Dxy rank correlation	0.407	0.362
Brier score	0.173	0.178

**Table 2:** *β*-Coefficient of predictive variables in the model with and without HbA1c.

	Model without HbA1c^1^	Model with HBA1c^1^
Previous baby ≥ 4 kg	102	1.01
Rgluc^2^ Rgluc^3^	1.99/ −2.1709	2.17/ −2.28
BMI^4^	0.05	0.05
HbA1c^1^	0.6464	
*R* ^2^ coefficient of determination	0.135	0.122
Somer's Dxy rank correlation	0.38	0.36
Harrell's c-index	0.69	0.68
Brier score	0.176	0.174
NRI^5^		
Categorical	0.0355 (*p* = 0.06034)	
Continuous	0.253 (*p* = 0.01607)	
IDI^6^	0.108 (*p* = 0.002)	

HbA1c^1^: glycated haemoglobin; Rgluc^2^: random glucose first spline; Rgluc^3^: random glucose second spline; BMI^4^: body mass index; NRI^5^: net reclassification index; IDI^6^: integrated discrimination improvement.

**Table 3:** Comparison of the efficacy of nomograms at probabilities of 10 and 15%.

Nomogram	With HbA1c^1^				Without HbA1c^1^			
	High risk		Low risk		High risk		Low risk	
Probability of GDM^2^	GDM^2^	No GDM^2^	GDM^2^	No GDM^2^	GDM^2^	No GDM^2^	GDM^2^	No GDM^2^
>10%	142 (25.6%)	354 (63.9%)	2 (0.4%)	56 (10.1%)	143 (25.8%)	361 (62.5%)	1 (0.2%)	49 (8.8%)
>15%	135 (24.3%)	304 (54.9%)	9 (1.6%)	115 (20.8%)	135 (24.3%)	316 (57.0%)	9 (1.6%)	94 (17%)

HbA1c^1^: glycated haemoglobin; GDM^2^: gestational diabetes.

**Table 4:** Comparison of the performance of scoring systems for the screening of GDM in our population.

Study	Risk factors	Risk calculation	AUROC^a^ (study population)	AUROC^a^ (original)	P^20^
Caliskan et al. [7]	(i) Age (years) (ii) Body mass index (BMI) (iii) Family history of diabetes mellitus (DM) (iv) Previous baby > 4000 g (v) Previous adverse pregnancy outcome	(i) <25 = 0; >25 = 1 (ii) <25 = 0; >25 = 1 (iii) No = 0; yes = 1 (iv) No = 0; yes = 1 (v) No = 0; yes = 1	0.5936 (0.5370–0.6504)	0.832 (0.793–0.867)	0.0005

Naylor et al. [8]	(i) Age (years) (ii) Body mass index (BMI) (iii) Race	(i) <30 = 0; 31–34 = 1; >35 = 2 (ii) <22 = 0; 22.1–25 = 2; >25.1 = 3 (iii) White/Black = 0; East Asian = 5; South Asian = 2	0.5897 (0.53214–0.64730)	0.733 (0.711–0.755)	0.0007

Van Leeuwen et al. [9]	(i) Body mass index (BMI) (ii) Race (iii) Family history of diabetes mellitus (FamHx) (iv) Gestational diabetes in previous pregnancy (GDMHx)	=1/[1 + exp(−*β*)] *β* = [−6.1 + (0.83 × non-Caucasian) + (0.57 × FamHx) − (0.67 × multipara no GDMhx) + (0.5 × multipara GDMhx) + (0.13 × BMI)] (Non-Caucasian: no = 0; yes = 1 Famhx: no = 0; yes = 1 Nullipara = 0; multiparaNoGDMhx = 1; multiparaGDMhx = 2)	0.5675 (0.51–0.63)	0.770 (0.690–0.850)	0.0015

Phaloprakarn et al. [10]	(i) Age (years) (ii) Body mass index (BMI) (iii) Family history of diabetes mellitus (FH) (iv) Previous baby > 4000 g (v) Previous adverse pregnancy outcome	6 age + 11 BMI + 109FH + 42 baby > 4000 g + 49 adverse pregnancy outcome ≥380 is positive screen	0.5182 (0.48664–0.54972)	0.769 (0.746–0.792)	<0.001

Teede et al. [11]	(i) Age (years) (ii) Body mass index (BMI) (iii) Race (iv) Family history of diabetes mellitus (v) Gestational diabetes in previous pregnancy	(i) <25 = 0; 25–34 = 1; >35 = 2 (ii) <20 = 0; 20–34.9 = 1; >35 = 2 (iii) White = 0; East Asian/South Asian/African = 1 (iv) No = 0; yes = 1 (v) No = 0; yes = 2	0.5863 (0.52934–0.64334)	0.703 (0.679–0.727)	0.0003

Harrison et al. [12]	(i) Age (years) (ii) Body mass index (BMI) (iii) Race (iv) Family history of diabetes mellitus (v) Gestational diabetes in previous pregnancies (vi) Fasting plasma glucose (FPG)	(i) <25 = 0; 25–34 = 1; >35 = 2 (ii) <20 = 0; 20–34.9 = 1; >35 = 2 (iii) White = 0; East Asian/South Asian/African = 1 (iv) No = 0; yes = 1 (v) No = 0; yes = 2 (vi) If score ≥ 3 assesses FPG			
Model 1		FPG 4.61–4.89 mmol/l	0.4751 (0.451–0.4995)	0.753 (0.675–0.832)	<0.001
Model 2		FPG ≥ 4.9 mmol/l	0.8662 (0.8336–0.89869)	0.83 (0.77–0.90)	0.1846

Syngekali et al. [13]	(i) Gestational diabetes in previous pregnancy (ii) Family history of DM (iii) Age (years) (iv) Weight (kg) (v) Height (cm) (vi) Race (vii) Method of conception (viii) Birth weight of previous pregnancy	No formula available in article	Could not be calculated as inadequate information on last pregnancy birth weight available	0.823 (0.820–0.826)	

Nanda et al. [14]	(i) Age (years) (ii) Body mass index (BMI) (iii) Race (iv) Gestational diabetes in previous pregnancy (v) Previous baby's birth weight > 90th centile (prevBW)	=1/[1 + exp(−*β*)] *β* = {−8.68947 + (0.05365 × age) + (0.10852 × BMI) + (1.00312 × South Asian) + (0.88785 × East Asian) + (3.72259 × previous GDM) + (0.67673 × prevBW > 90th centile)}	0.6218 (0.56301–0.68058)	0.788 (0.759–0.817)	<0.001

Capula et al. [15]	(i) Age (years) (ii) Pregravid body mass index (BMI) (iii) Previous gestational diabetes (GDM), polycystic ovarian syndrome (PCOS) (iv) Fasting plasma glucose (FPG) 5.6–6.9 mmol/l before pregnancy	= Constant − 2.2532 × (age/10) + 0.4128 × (age/10)^2^ + 0.0795 × pregravid BMI Constant is intercept depending on previous GDM, PCOS, FPG 5.6–6.9 mmol/l before pregnancy	0.5337 (0.48 0.59)	(Information not available)	

^a^AUROC: area under receiver operating curve.
