# Supplementary material for: Selective Screening Strategies for Gestational Diabetes: A Prospective Cohort Observational Study
Source: J Diabetes Res. 2017 Oct 22;2017:2849346. doi: 10.1155/2017/2849346 (PMC5671730; doi:10.1155/2017/2849346)
Supplement: Supplementary file 1 — Figure S1. Odds ratio of full model (continuous variables: 75th versus 25th percentile). Figure S2 Correcting the model for optimism. Figure S3. Comparison of predictive value of model with and without HbA1c. [file 2849346.f1.docx]

**S1 Odds ratio of full model (continuous variables: 75^th^ versus 25^th^ percentile)**


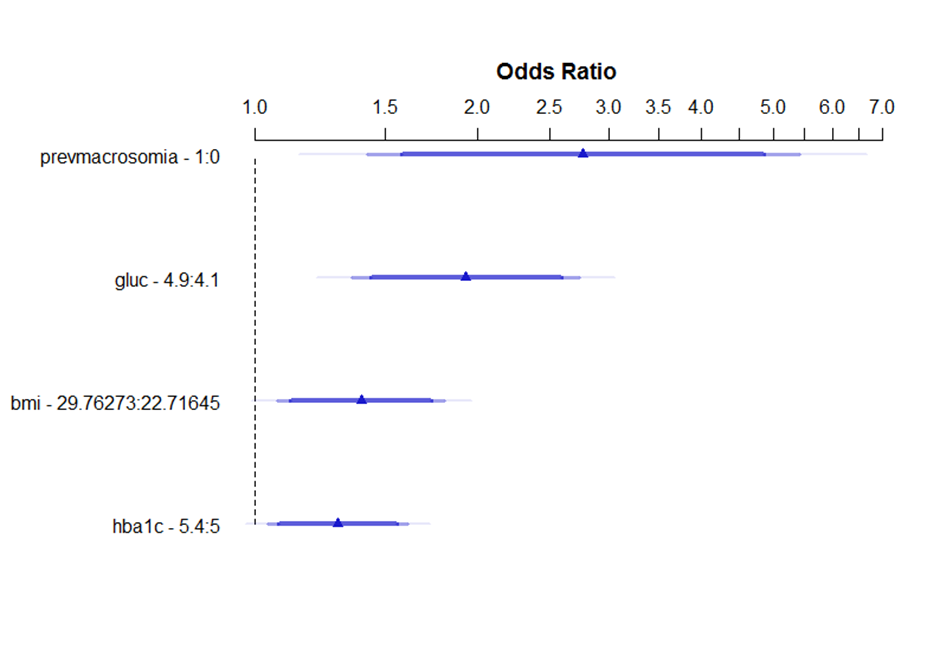


**S2 Correcting the model for optimism**


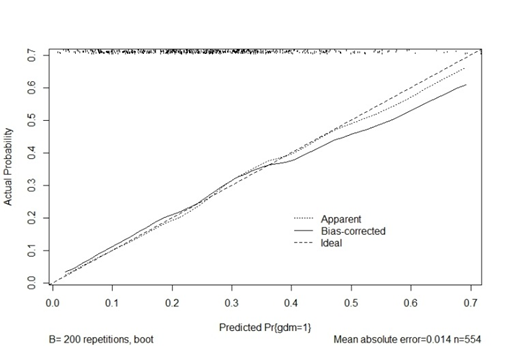


**S3 Comparison of predictive value of model with and without HbA1c**

**
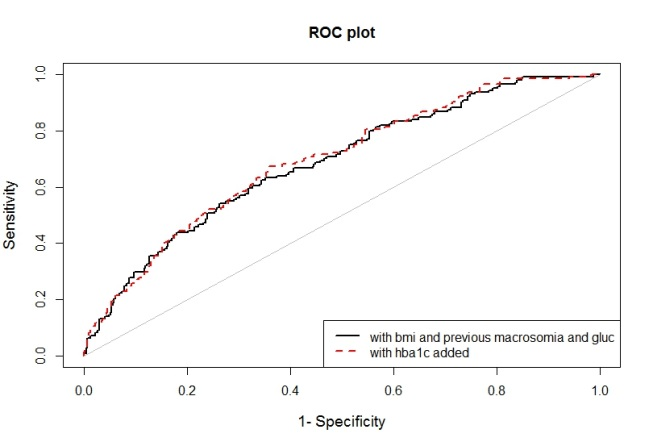
**

| **Discrimination Index** | | **Without HbA1c** | **With HbA1c** |
| --- | --- | --- | --- |
| **Harrel’s C index** | | 0.69 | 0.68 |
| **Brier score** | | 0.176 | 0.174 |
| **Net reclassification index (NRI)** | **Categorical** | 0.0355 (-0.0015 – 0.0725); p-0.06034 | |
|  | **Continuous** | 0.253 (0.0659 – 0.4401); p-0.01607 | |
| **Integrated discrimination improvement (IDI)** | | 0.108 (0.002 – 0.0195); p-0.01607 | |
